# Delivery of a baby with severe combined immunodeficiency at 31 weeks gestation following an extreme preterm prelabour spontaneous rupture of the membranes: a case report

**DOI:** 10.1186/1752-1947-3-118

**Published:** 2009-11-12

**Authors:** Sally J Watkinson, Christopher CT Lee, Christopher V Steer

**Affiliations:** 1 Department of Obstetrics and Gynaecology, Princess Royal University Hospital, Farnborough, Kent, UK

## Abstract

**Introduction:**

If left untreated, severe combined immunodeficiency can lead to an acute susceptibility to infection. The intrauterine environment is sterile until the amniotic membranes rupture. The vaginal flora then ascends into the genital tract, thus increasing the risk of chorioamnionitis. An extremely premature and prolonged membrane rupture is associated with a dismal prognosis for an immunocompetent preterm fetus. There are no case reports to date that detail the outcome of an immunocompromised preterm baby following prolonged rupture of membranes.

**Case presentation:**

We present the case of a 32-year-old Indian woman who delivered a 31-week gestational baby who had a severe combined immunodeficiency following premature prelabour prolonged rupture of the membranes at the 14^th ^week of gestation.

**Conclusion:**

Extreme preterm prelabour spontaneous rupture of membranes in an underlying condition of severe combined immunodeficiency does not necessarily lead to an unfavourable outcome.

## Introduction

Severe combined immunodeficiency (SCID) is a combined cellular and humoral immunodeficiency resulting from a lack of functional T and B lymphocytes. In some cases, SCID is also combined with a deficiency of natural killer cells. This condition is extremely rare, affecting approximately only 1 in 100,000 live births. SCID is usually diagnosed after the 3^rd ^month of gestation, during the onset of one or more serious infections such as recurrent or persistent infections despite conventional treatment, infections with opportunistic organisms such as Pneumocystis, and a failure to thrive. SCID is usually X-linked and can be diagnosed through genetic testing.

Babies in general are more susceptible to infections as compared to adults. This susceptibility is even more pronounced in preterm babies and those who have been potentially exposed to maternal flora following a breach in the amniotic membrane due to a prolonged prelabour spontaneous rupture of the membranes (SROM). Pathogens gaining entry into the baby's system through the mucosa of the respiratory and gastrointestinal tracts are poorly localised. The preterm baby can thus easily become systemically unwell.

The sterile environment of the intrauterine amniotic sac limits the need for learned immune responses to specific antigens prior to birth. Upon birth, a normal baby has some immunoglobulins (Ig), with IgG as predominant because it is small enough to cross the placenta and be transferred from the mother. The level of IgG at birth is similar to that of the mother and provides passive immunity to mainly viral infections in the first few months of life.

Meanwhile, IgM and IgA do not cross the placental barrier but are produced by the normal fetus *in utero *from approximately 28 weeks of gestation. Levels of IgM at term are 20% of those present in adults, unless intrauterine infection develops and the fetus mounts an immune response to further elevate IgM levels. IgM provides a degree of protection to the neonate from enteric infections. While IgA levels are very low at birth, its production increases rapidly following delivery to reach adult values within two months. IgA protects against infection of the respiratory tract, the gastrointestinal tract and the eyes. The levels of both IgM and IgG at birth are lower in preterm than in term neonates [1].

There is no effect to the immune function of a female carrier of X-linked SCID. Thus, the fetus of such a woman generally has normal IgG levels *in utero *and at birth.

The prognosis for a normal pregnancy where the membranes rupture at 14 weeks is dismal due primarily to the risk of miscarriage secondary to infection. Even with appropriate treatment, approximately 50% of pregnancies are delivered each subsequent week following preterm SROM. Therefore, when the membranes rupture before 20 weeks of gestation the probability of reaching viability is <5% [2].

A second reason for dismal prognosis is the risk of neonatal death secondary to pulmonary hypoplasia when pregnancy becomes viable. The chance of pulmonary hypoplasia is lessened if the fluid re-accumulates before 24 weeks of gestation. One study using a multivariate analysis suggested that the likelihood for neonate survival increases by 2.7 (95% CI 1.45 to 4.65) for every 5-mm increase in the depth of amniotic fluid during the follow-up from rupture up to the 24^th ^week of gestation [3]. Despite dismal prognosis, however, expectant management for preterm SROM at 14 weeks may be appropriate if the mother is well-informed of the risks for the neonate.

The conclusions of the ORACLE trials [4,5] indicated that a course of oral erythromycin is the antibiotic of choice in the treatment of expectantly managed preterm SROM. This is because erythromycin is associated with a reduction in neonatal infection, slight prolongation of pregnancy with no increase in the likelihood of developing necrotising enterocolitis. Based on evidence from a study of preterm SROM conducted by the Maternal-Fetal Medicine Units of the National Institute of Child Health and Human Development, 7 days of antibiotics should be prescribed because longer courses have not been shown to be more effective and may actually promote antibiotic resistance [6].

Maternal protein C deficiency is associated with an increased risk of poor pregnancy outcome including miscarriage, stillbirth and preterm delivery.

## Case presentation

A 32-year-old Indian woman presented to our gynaecology clinic with secondary subfertility. Routine screening found her to be positive for protein C deficiency. In 1999, the woman had previously delivered a son by elective caesarean section following a diagnosis of transverse lie at 39 weeks of gestation. The baby was subsequently diagnosed with SCID and died at 8 months due to this condition. A specialist genetic centre indicated that this was an X-linked condition and the woman is a carrier of the mutation c.283G>A (W90X) in exon 2 of her IL2RγC gene.

She was advised of a 50%-risk of any subsequent male offspring being affected by SCID and was thus offered preimplantation genetic diagnosis. However she subsequently conceived the index pregnancy spontaneously. She was commenced on aspirin, low molecular weight heparin (LMWH) and progesterone pessaries as soon as the pregnancy was confirmed by ultrasound at 6 weeks. Resutls of her nuchal translucency screening and first trimester anomaly scan were normal.

The woman presented with leakage of liquor at 14^+5 ^weeks gestation. Spontaneous rupture of the membranes was confirmed clinically and through an ultrasonography. She was commenced on antibiotic prophylaxis with oral erythromycin and her medication of progesterone pessaries was discontinued. Serial specialist ultrasonography at 16, 18, 20 and 22 weeks confirmed a normally grown male fetus with no obvious structural defects. The placenta was posterior and low, and the severe oligohydramnios was persistent. In view of the oligohydramnios due to extreme preterm SROM, an invasive genetic testing was not felt to be appropriate. The risk of this male fetus being affected with SCID therefore remained at 50%. Because of the extremely poor prognosis for the baby, the couple was offered a termination of pregnancy but they declined. The pregnancy continued, septic markers remained negative, and she remained on continuous oral erythromycin, aspirin and LMWH.

At 24^+4 ^weeks gestation, the woman self-referred with a history of unprovoked vaginal bleeding. She was given dexamethasone and was transferred to a centre with Level 1 neonatal facilities. She stayed at the centre until her transfer back to her local hospital at 28^+5^. Erythromycin was discontinued at 25^+6 ^weeks gestation, maternal clinical and laboratory signs of infection remained absent, and ultrasound scan at 28 weeks showed normal growth of the fetus and an amniotic fluid index of 8.4 cm. Upon readmission, she was recommenced on erythromycin and discharged from the hospital. She was advised instead to visit the antenatal day unit twice a week for regular assessments.

The woman remained stable until she presented again at 31^+4 ^weeks with lower abdominal pain and recurrent slight vaginal bleeding. It was found that her cervix was dilating and that the baby's presentation was breech. An emergency caesarean section was thus performed under spinal anaesthetic. She made a good postoperative recovery and was discharged after 5 days.

The baby boy was born with Apgar scores of 6^1 ^and 10^5 ^minutes and weighed 1830 g (50^th ^percentile). He required some initial resuscitation but was transferred to our special care baby unit with spontaneous respiratory effort in facial oxygen. His white cell count and C-reactive protein level were within the normal range (6.3 and <5, respectively) while his blood cultures were negative at birth. The baby was treated with oral nystatin, intravenous benzyl penicillin and gentamicin. Isolation and barrier nursing was also advised.

X-linked SCID (consistent with the mother's carrier type) was confirmed by genetics testing during the neonatal period. He received intravenous therapy of 1 g immunoglobulin on Day 3 and was transferred to a specialist paediatric centre on Day 6. The baby underwent an unconditioned CD34-selected mismatched family donor bone marrow transplant (from his father) on Day 46. He continued to receive monthly 2 g intravenous immunoglobulin and regular outpatient specialist paediatric immunology reviews. As of this writing, the baby is thriving and still breastfeeding at 1 year of life. He is also showing a good response to his treatment.

## Discussion

Fetuses affected by SCID have significantly lower levels of IgM and IgA at birth compared to gestationally age-matched immunocompetent babies. It is thus likely that SCID affected babies will be unable to mount any IgA and IgG immune response *in utero *in response to ascending infection as a result of SROM. As immunocompetent premature babies produce such a poor response *in utero*, the question is whether SCID affected babies in a condition of preterm SROM have a higher risk of *in utero *infection than those who do not have SCID.

## Conclusion

This baby was born in good condition and is currently thriving and living a normal life. This unusual case of adverse prognostic factors, including an underlying genetic condition, prelabour preterm SROM and maternal protein C deficiency, demonstrates that the outcome for babies with this condition is not necessarily hopeless.

This baby was born without evidence of *in utero *infection despite the expected poor prognosis of having premature prelabour SROM from 14+ weeks. Had an infection occurred, the prognosis would have been certainly poor. However, this would not be as a result of the SCID condition, and the baby would have had normal levels of IgG at birth because of transplacental transfer from his mother. Because normal babies have a poor IgA and IgM immune response *in utero *at 31 weeks, it is very unlikely that the inability to mount any IgA or IgM response because of the SCID condition had any effect on the outcome in this case. It is therefore reasonable to conclude that the *in utero *management of fetuses with known or suspected primary congenital immunodeficiency including SCID should not be managed any differently than preterm prelabour SROM in normal pregnancies.

The counselling of parents regarding the possible prognosis following a diagnosis of SROM at extreme prematurity should not be altered if the baby is known or suspected to be affected with SCID. With regard to the antenatal use of antibiotics in babies with SCID, the standard dose and duration of treatment of erythromycin 250 mg 4 times daily for 7 days should be prescribed. Measures to discourage ascending infection such as the avoidance of vaginal examinations and sexual intercourse should be advised regardless of an underlying diagnosis of SCID.

## Abbreviations

SCID: severe combined immunodeficiency; SROM: spontaneous rupture of the membranes; Ig: immunoglobulins; LMWH: low molecular weight heparin.

## Competing interests

The authors declare that they have no competing interests.

## Consent

Written informed consent was obtained from the patient for publication of this case report and any accompanying images. A copy of the written consent is available for review by the Editor-in-Chief of this journal.

## Authors' contributions

SW and CL wrote the article. CS was the lead clinician in charge of the patient's care. All authors read and approved the final manuscript.
